# Prognostic and predictive value of androgen receptor expression in postmenopausal women with estrogen receptor-positive breast cancer: results from the Breast International Group Trial 1–98

**DOI:** 10.1186/s13058-019-1118-z

**Published:** 2019-02-22

**Authors:** Kevin H. Kensler, Meredith M. Regan, Yujing J. Heng, Gabrielle M. Baker, Michael E. Pyle, Stuart J. Schnitt, Aditi Hazra, Roswitha Kammler, Beat Thürlimann, Marco Colleoni, Giuseppe Viale, Myles Brown, Rulla M. Tamimi

**Affiliations:** 1 Department of Medical Oncology, Dana-Farber Cancer Institute, Boston, MA USA; 2 Department of Epidemiology, Harvard T.H. Chan School of Public Health, Boston, MA USA; 3 Department of Biostatistics and Computational Biology, Dana-Farber Cancer Institute, Harvard Medical School, Boston, MA USA; 4 Department of Pathology, Beth Israel Deaconess Medical Center, Boston, MA USA; 5 Department of Pathology, Brigham and Women’s Hospital, Boston, MA USA; 6 Department of Medicine, Brigham and Women’s Hospital, Boston, MA USA; 7 International Breast Cancer Study Group Coordinating Center, Central Pathology Office, Bern, Switzerland; 8 Breast Center, Cantonal Hospital, St. Gallen and SAKK, Berne, Switzerland; 9 Division of Medical Senology, IEO, European Institute of Oncology IRCCS, Milan, Italy; 10 Department of Pathology, IEO, European Institute of Oncology IRCCS, University of Milan, Milan, Italy; 11 Channing Division of Network Medicine, Brigham and Women’s Hospital and Harvard Medical School, Boston, MA USA

**Keywords:** Androgen receptor, Breast cancer, Letrozole, Tamoxifen, BIG 1–98

## Abstract

**Background:**

The androgen receptor (AR) is an emerging prognostic marker and therapeutic target in breast cancer. AR is expressed in 60–80% of breast cancers, with higher prevalence among estrogen receptor-positive (ER+) tumors. Androgen treatment inhibits ER signaling in ER+/AR+ breast cancer cell lines, and AR expression is associated with improved survival for this subtype in epidemiologic studies. However, whether AR expression modifies the efficacy of selective ER modulators or aromatase inhibitors for ER+ cancers remains unclear.

**Methods:**

We evaluated the prognostic and predictive value of AR expression among 3021 postmenopausal ER+ breast cancer patients in the Breast International Group (BIG) trial 1–98. The BIG 1–98 study was a four-armed, double-blind, phase III randomized clinical trial that compared 5 years of tamoxifen or letrozole monotherapy, or sequences of 2 years and 3 years treatment with one drug and then the other. AR expression was measured by immunohistochemistry and the percentage of AR-positive nuclei was quantified. The association between AR expression and prognosis was evaluated using Cox proportional hazards models. Continuous AR-by-treatment interactions were assessed using Subpopulation Treatment Effect Pattern Plots (STEPP).

**Results:**

Eighty-two percent of patients had AR+ (≥ 1%) tumors. Patients with AR+ cancers were more likely to have smaller, lower-grade tumors, with higher expression of ER and PR. AR expression was not associated with breast cancer-free interval (BCFI) (415 events) over a median 8.0 years of follow-up (*p* = 0.12, log-rank test). In multivariable-adjusted models, AR expression was not associated with BCFI (HR = 1.07, 95% CI 0.83–1.36, *p* = 0.60). The letrozole versus tamoxifen monotherapy treatment effect did not significantly differ for AR+ tumors (HR = 0.63, 95% CI 0.44–0.75, *p* = 0.003) and AR− tumors (HR = 0.39, 95% CI 0.21–0.72, *p* = 0.002) (*p*-heterogeneity = 0.16). STEPP analysis also suggested no heterogeneity of the treatment effect across the continuum of AR expression.

**Conclusions:**

AR expression was not associated with prognosis, nor was there heterogeneity of the letrozole versus tamoxifen treatment effect by AR expression. These findings suggest that AR expression may not be an informative biomarker for the selection of adjuvant endocrine therapy for postmenopausal women with ER+ breast cancers.

**Trial registration:**

ClinicalTrials.gov, NCT00004205, Registered 27 January 2003—Retrospectively registered, https://clinicaltrials.gov/ct2/show/study/NCT00004205.

**Electronic supplementary material:**

The online version of this article (10.1186/s13058-019-1118-z) contains supplementary material, which is available to authorized users.

## Background

Sex steroid hormone receptor signaling is vital in the development and progression of breast cancers. The role of the estrogen receptor (ER) in breast cancer has been well elucidated, and estrogen blockade using selective estrogen receptor modulators (SERMs) and aromatase inhibitors is fundamental in breast cancer therapeutics [1, 2]. The androgen receptor (AR) is expressed in 60–80% of breast tumors, though its prevalence is correlated with tumor ER expression [3–7]. Approximately 90% of ER+ breast cancers express AR, while only 20–30% of ER− breast cancers are AR+.

Preclinical and clinical data support differential effects of AR signaling in breast cancers, dependent on tumor ER expression [8–10]. In ER− breast cancers, AR signaling can drive tumor growth [11]; however, epidemiologic studies have yielded inconsistent evidence regarding the prognostic value of AR in this subtype [12]. In contrast, AR signaling has been shown to antagonize cell proliferation in ER+ breast cancers in in vitro models [13]. This may occur through competition between AR and ER for binding sites at estrogen response elements or through competition for transcriptional co-regulators [13]. Population-based studies support the hypothesis that AR may antagonize the detrimental effects of ER signaling, and have shown a consistent inverse association between tumor AR expression and mortality among patients with ER+ breast cancer [7, 12, 14].

The Breast International Group (BIG) trial 1–98 was a phase III randomized clinical trial that evaluated letrozole (an aromatase inhibitor) versus tamoxifen (a SERM) as an adjuvant therapy for early stage hormone receptor-positive breast cancer among 8010 postmenopausal women [15]. The initial and subsequent reports found superior disease-free survival among those randomized to 5 years of letrozole monotherapy compared to those randomized to tamoxifen monotherapy [16, 17]. Secondary analyses found that the letrozole versus tamoxifen treatment effect was stronger for tumors with high Ki67 expression, but the effect did not differ by tumor progesterone receptor (PR) or human epidermal growth factor 2 (HER2) expression [18–20].

There is conflicting evidence surrounding AR as a predictive marker of tamoxifen response, with prior studies indicating that high AR expression is associated with improved response and that a high AR:ER ratio is associated with tamoxifen resistance [21, 22]. No studies to date have evaluated AR as a predictor of response to letrozole. Given the purported antagonistic effects of AR signaling in ER+ breast cancer, we postulate that the efficacy of letrozole may be stronger in AR+/ER+ cancers. Blockade of testosterone conversion to estradiol may result in increased AR signaling and subsequently inhibit tumor growth. Androgen therapies were effective for the treatment of hormone-responsive breast cancers and were commonly administered prior to the introduction of tamoxifen [23, 24]. Additionally, cotreatment with bicalutamide, a nonsteroidal AR antagonist, eliminated the antiproliferative effects of letrozole in MCF7 cells [25].

We leveraged the resources of the BIG 1–98 trial to evaluate AR protein expression as a marker of prognosis among postmenopausal women with early stage ER+ breast cancer. Additionally, we also assessed AR expression as a predictor of differential efficacy of letrozole and tamoxifen adjuvant therapy in this population.

## Methods

### Study design

BIG 1–98 was an international, multicenter, phase III, double-blind, four-arm randomized trial conducted among postmenopausal women with early stage hormone receptor-positive breast cancer. The design of the trial has been described in detail previously [15–17]. From March 1998 to March 2000, participants were randomized to receive 5 years of monotherapy with either letrozole (2.5 mg/d) or tamoxifen (20 mg/d). Participants enrolled between April 2000 and May 2003 were randomized to either 5 years monotherapy with either letrozole or tamoxifen, 2 years of letrozole followed by 3 years of tamoxifen, or 2 years of tamoxifen followed by 3 years of letrozole. The primary efficacy analysis included 8010 eligible participants and showed superiority of letrozole therapy to tamoxifen therapy [16]. Following publication of the primary efficacy analysis, participants in the tamoxifen monotherapy arm were offered the opportunity to receive letrozole and 619 elected to cross over. All patients provided informed written consent, and all study protocols were approved by ethics committees at each study center and relevant health authorities. BIG 1–98 is registered with ClinicalTrials.gov (identifier NCT00004205).

### Tissue markers

Tumor tissue biospecimens were collected retrospectively in accordance with institutional guidelines and national laws, with the financial support of Novartis. Tissue blocks and/or slides were obtained for 6549 participants at the International Breast Cancer Study Group (IBCSG) Central Pathology Office in Milan, Italy. For 3784 participants, two 1-mm-diameter cores per participant were obtained from their formalin-fixed paraffin-embedded tumor specimens and assembled into tissue microarrays (TMAs) in Italy.

AR was centrally evaluated by immunohistochemistry using the Dako AR441 antibody (1:200 dilution) at the Dana-Farber/Harvard Cancer Center Specialized Histopathology Services Core, Boston, MA, USA (TMA sectioning and antibody staining) and Beth Israel Deaconess Medical Center, Boston, MA, USA (scoring). The percentage of positive epithelial nuclei was estimated using Definiens Tissue Studio automated imaging analysis software version 4.4.2 (Munich, Germany). The average of AR expression across the two cores was calculated for each participant weighted by the number of detected nuclei. A cutoff of 1% expression or greater was used to define AR positivity in primary analyses. The Spearman correlation between AR expression measured by Definiens and three-tier manual pathologist scoring (< 1%, 1–10%, > 10%) was 0.61 in a test TMA (*n* = 42 individuals). Expression of ER, PR, and Ki67 was previously evaluated on whole sections by immunohistochemistry (IHC) at the IBCSG Central Pathology Office, as was the expression of HER2 by IHC and fluorescent in situ hybridization [18–20]. ER and PR expression were scored in increments of 5%, and Ki67 expression was scored as a continuous measure. All tissue marker assays were conducted blinded to participants’ treatment assignment and outcome. Spearman correlations were 0.24 between AR and ER expression, 0.16 between AR and PR expression, and − 0.02 between AR and Ki67 expression.

### Endpoints

The primary endpoint in this analysis was invasive breast cancer-free interval (BCFI), which was defined in concordance with STEEP criteria as the time from randomization until the earliest of invasive local-regional or distant recurrence or new invasive breast cancer in the contralateral breast [26]. Disease-free survival (DFS) was evaluated as a secondary endpoint and defined as time from randomization until the earliest of invasive recurrence, new invasive breast cancer in the contralateral breast, second (non-breast) invasive malignancy, or death from any cause.

### Analytic population

**Fig. 1:**
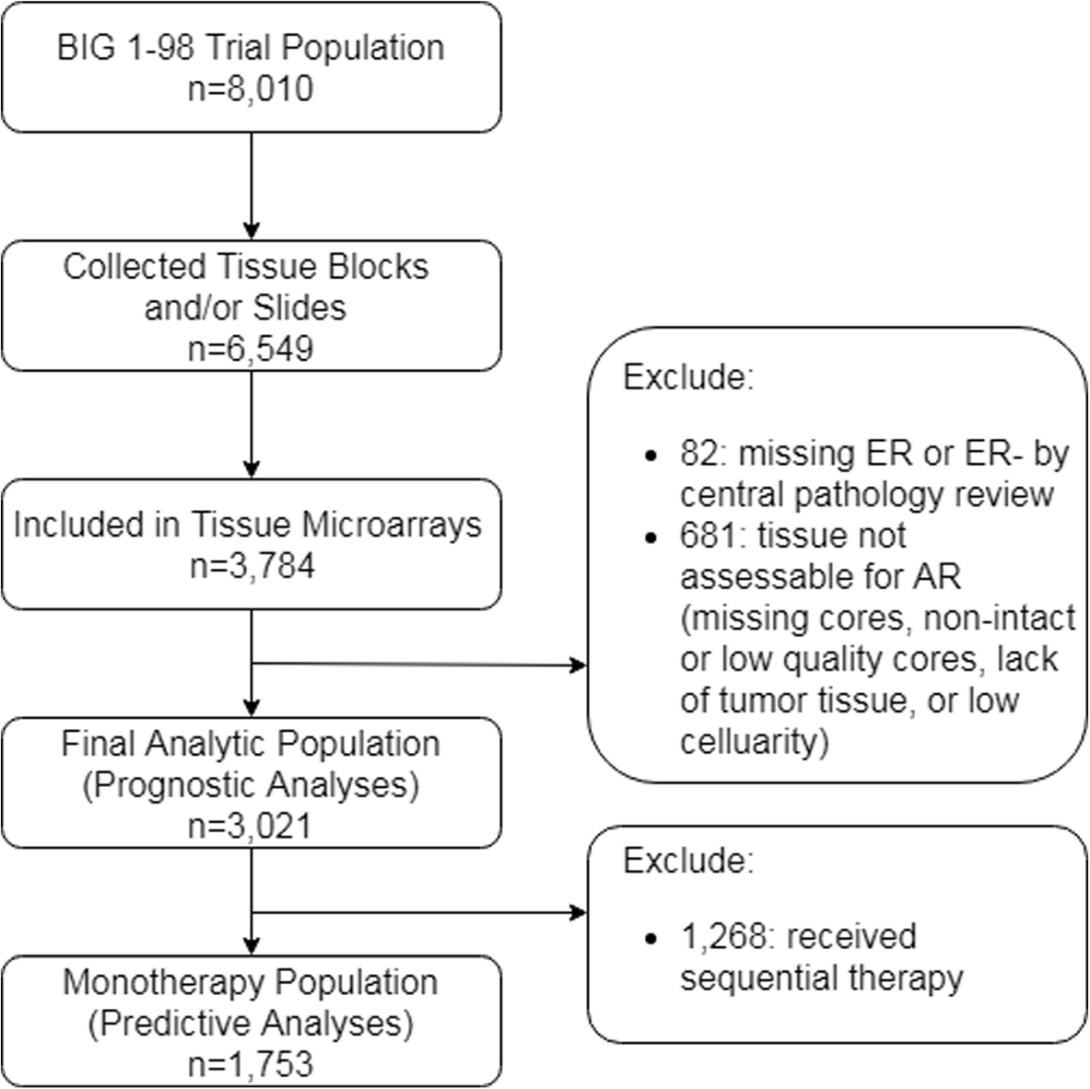
Flow diagram of participant inclusion

A comparison of trial participants with and without assessable AR expression is shown in Additional file 1: Table S1. Of the 3784 participants whose specimens were assembled on the TMAs, 82 participants did not have ER expression assessed or were assessed as ER- by central pathology review and were excluded, and 681 participants’ tissues were not assessable for AR staining due to missing cores, non-intact or poor quality cores, lack of tumor tissue in the core, or low cellularity, resulting in an analytic population of 3021 women (Fig. 1). Of these, 1753 were assigned to either letrozole or tamoxifen monotherapy. Prognostic analyses included participants from the monotherapy and mixed therapy arms (*n* = 3021), while predictive analyses were limited to the monotherapy population (*n* = 1753).

### Statistical analysis

The associations between AR expression and patient and tumor characteristics were evaluated using chi-square tests for categorical variables and Wilcoxon rank sum tests for continuous variables. The distribution of BCFI and DFS were estimated using the Kaplan-Meier method. The association between AR expression and BCFI and DFS was evaluated both by the log-rank test and using Cox proportional hazards models to estimate hazard ratios (HRs) and 95% confidence intervals (95% CIs). Models for this association were stratified by randomized treatment assignment and receipt of chemotherapy, and were adjusted for participant, tumor, and local treatment covariates. AR expression was evaluated both as a dichotomous variable (≥1% versus < 1%) and as a continuous variable. Restricted cubic splines were used to evaluate potential non-linearity of the association between AR expression and BCFI and DFS, though tests for non-linearity were highly non-significant, and AR expression was therefore modeled linearly as a continuous variable [27]. ER, PR, and Ki67 were included as continuous linear measures in analyses.

AR was assessed as a predictive biomarker using Cox proportional hazards models. In these analyses, participants who selectively crossed over from tamoxifen monotherapy to letrozole monotherapy were censored at the time of crossover. Heterogeneity of the treatment effect by dichotomous AR expression was assessed using a likelihood ratio test for an AR-by-treatment interaction. Subpopulation Treatment Effect Pattern Plot (STEPP) analysis was used to evaluate heterogeneity of the treatment effect across the continuum of AR expression [28, 29]. The STEPP approach entails calculating treatment effects at 5 years post-randomization within sliding subpopulations defined by AR expression. Each subpopulation contained approximately 200 patients and shared approximately 100 patients with neighboring subpopulations. STEPP was used to estimate 5-year cumulative incidences of recurrence in a competing risk model and hazard ratios. All analyses were conducted using R version 3.5. All statistical tests were two-sided, and *p* < 0.05 was considered statistically significant.

For evaluation of AR as a prognostic marker, with a sample size of 3021 women, 415 events, 82% AR+ tumors, and setting *α* = 0.05 for a two-sided test, there was 80% power to detect an HR of 0.69 or lower for BCFI [30]. To evaluate the AR-by-treatment interaction, with 1753 women in the monotherapy population, 236 events, 83% AR+ tumors in the letrozole arm, 80% AR+ tumors in the tamoxifen arm, and setting α = 0.05 for a two-sided test, there was 80% power to detect a HR of 0.38 or lower for the AR-by-treatment product term for BCFI [31].

## Results

Distributions of participant, tumor, and treatment variables are shown in Table 1. Eighty-two percent of breast cancers in this population were AR+ (≥ 1%) and the median AR expression was 12.5% (interquartile range [IQR] 2.1–30.6%). Women with AR+ breast cancers were more likely to have smaller tumors (*p* < 0.001) and lower histologic grade tumors (*p* < 0.001). Correspondingly, women with AR+ cancers were more likely to have received breast conserving surgery (*p* < 0.001), but receipt of chemotherapy did not differ by AR status (*p* = 0.21). AR+ tumors had higher ER and PR expression than AR− tumors (both *p* < 0.001), but were less likely to overexpress HER2 (*p* = 0.04). Ki67 expression did not significantly differ between AR+ and AR− tumors (*p* = 0.07).

**Table 1:** Study population characteristics by tumor AR expression

Characteristic	AR positive^a^ (*n* = 2475)	AR negative^a^ (*n* = 546)	*P* value^b^
Treatment assignment, %			–
Tamoxifen	21	21	
Letrozole	22	18	
Tamoxifen ➔ Letrozole	21	21	
Letrozole ➔ Tamoxifen	21	20	
Tamoxifen (2-arm period)	7	12	
Letrozole (2-arm period)	8	10	
Received chemotherapy, %	29	32	0.21
Local therapy, %			< 0.001
Breast conserving surgery with radiation therapy	64	55	
Breast conserving surgery without radiation therapy	4	5	
Mastectomy with radiation therapy	12	18	
Mastectomy without radiation therapy	20	22	
Age at randomization, mean (SD)	62 (8)	62 (8)	0.10
Tumor size, %			< 0.001
≤ 2 cm	67	54	
> 2–< 5 cm	29	41	
≥ 5 cm	3	4	
Missing	1	1	
Number of lymph nodes positive, %			0.15
0	63	58	
1–3	26	30	
4–9	7	8	
10+	3	3	
Not evaluable	1	1	
Tumor grade, %			< 0.001
1	22	14	
2	57	50	
3	21	36	
Missing	0	1	
ER expression (%), median (25th–75th percentile)	95 (90–99)	90 (75–95)	< 0.001
PR expression (%), median (25th–75th percentile)	70 (20–90)	50 (5–84)	< 0.001
Missing, *n*	21	8	
Ki67 expression (%), median (25th–75th percentile)	12 (6–18)	12 (6–20)	0.07
Missing, *n*	59	31	
HER2 expression, %			0.04
Positive	5	8	
Negative	95	92	
Missing	0	0	

^a^AR expression is defined as ≥ 1% positive, < 1% negative

^b^Hypothesis testing for differences in distributions between AR+ and AR− tumors used chi-square and Wilcoxon rank sum tests for categorical and continuous endpoints, respectively. Design variables (two- or four-arm randomization period and treatment assignment) were not compared. Individuals with missing data were excluded when performing hypothesis tests for continuous variables

**Fig. 2:**
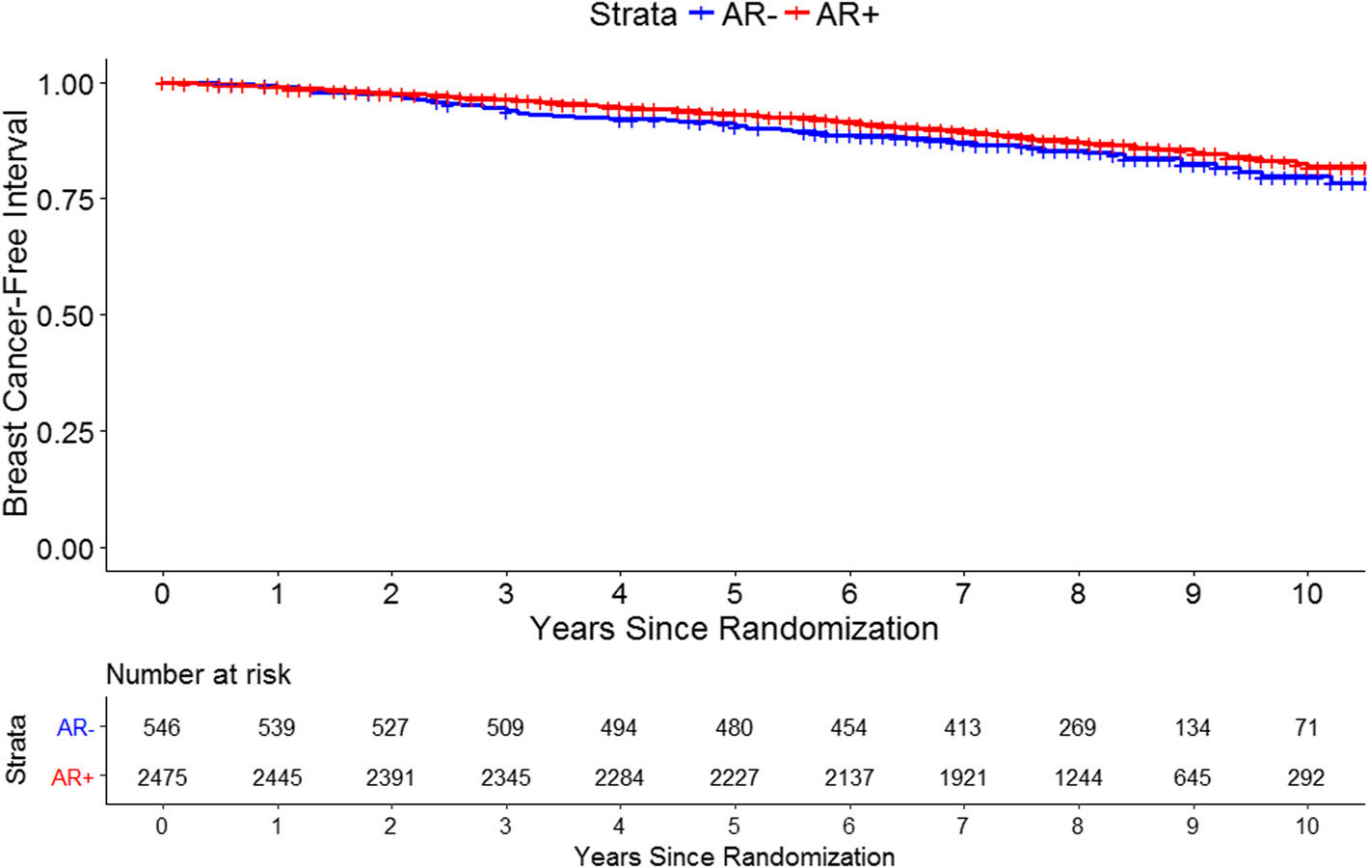
Kaplan-Meier estimate of breast cancer-free interval by tumor AR expression. AR expression is defined as ≥ 1% positive, < 1% negative. *P* value from log-rank test is 0.12

Over a median of 8.0 years follow-up (IQR 7.0–9.0 years) after randomization, a total of 415 BCFI events (14% of analytic population) and 676 DFS events (22%) occurred. Five-year BCFI was 93.1% (95% CI 92.1–94.2%) among women with AR+ tumors and 90.5% (95% CI 88.1–93.0%) among women with AR− tumors, though differences in BCFI were not significant (*p* = 0.12, log-rank test) (Fig. 2). There was also no statistically significant difference in DFS by AR status (*p* = 0.35) (Additional file 1: Fig. S1). After adjustment for participant, tumor, and treatment covariates, tumor AR expression was not associated with BCFI (HR = 1.07, 95% CI 0.83–1.38, *p* = 0.60) (Table 2), nor was it associated with DFS (HR = 1.12, 95% CI 0.91–1.38, *p* = 0.27). When evaluating AR expression continuously, each successive 10% increase in AR expression was not associated with BCFI (HR = 1.02, 95% CI 0.97–1.08, *p* = 0.50).

**Table 2:** Multivariable-adjusted hazard ratios (95% CI) for breast cancer-free interval and disease-free survival by tumor AR expression

			AR+ vs. AR−	10% increase
	Individuals	Events	HR (95% CI)	HR (95% CI)
Breast cancer-free interval				
Model 1^a^	3021	415	0.84 (0.66–1.07)	0.97 (0.92–1.02)
			*p* = 0.16	*p* = 0.26
Model 2^b^	2907	398	1.07 (0.83–1.39)	1.02 (0.97–1.08)
			*p* = 0.60	*p* = 0.50
Disease-free survival				
Model 1	3021	676	0.95 (0.78–1.15)	0.97 (0.93–1.01)
			*p* = 0.58	*p* = 0.19
Model 2	2907	650	1.12 (0.91–1.38)	1.01 (0.96–1.05)
			*p* = 0.27	*p* = 0.77

Hazard ratios (HRs) compare AR+ (≥ 1%) to AR− (< 1%) cancers or represent association per each successive 10% increase in AR expression

^a^Model 1: Stratified by treatment assignment and receipt of chemotherapy, and adjusted for age at randomization

^b^Model 2: Model 1 + adjusted for tumor size, lymph node involvement, local therapy, ER, PR, HER2, and Ki67 expression

**Fig. 3:**
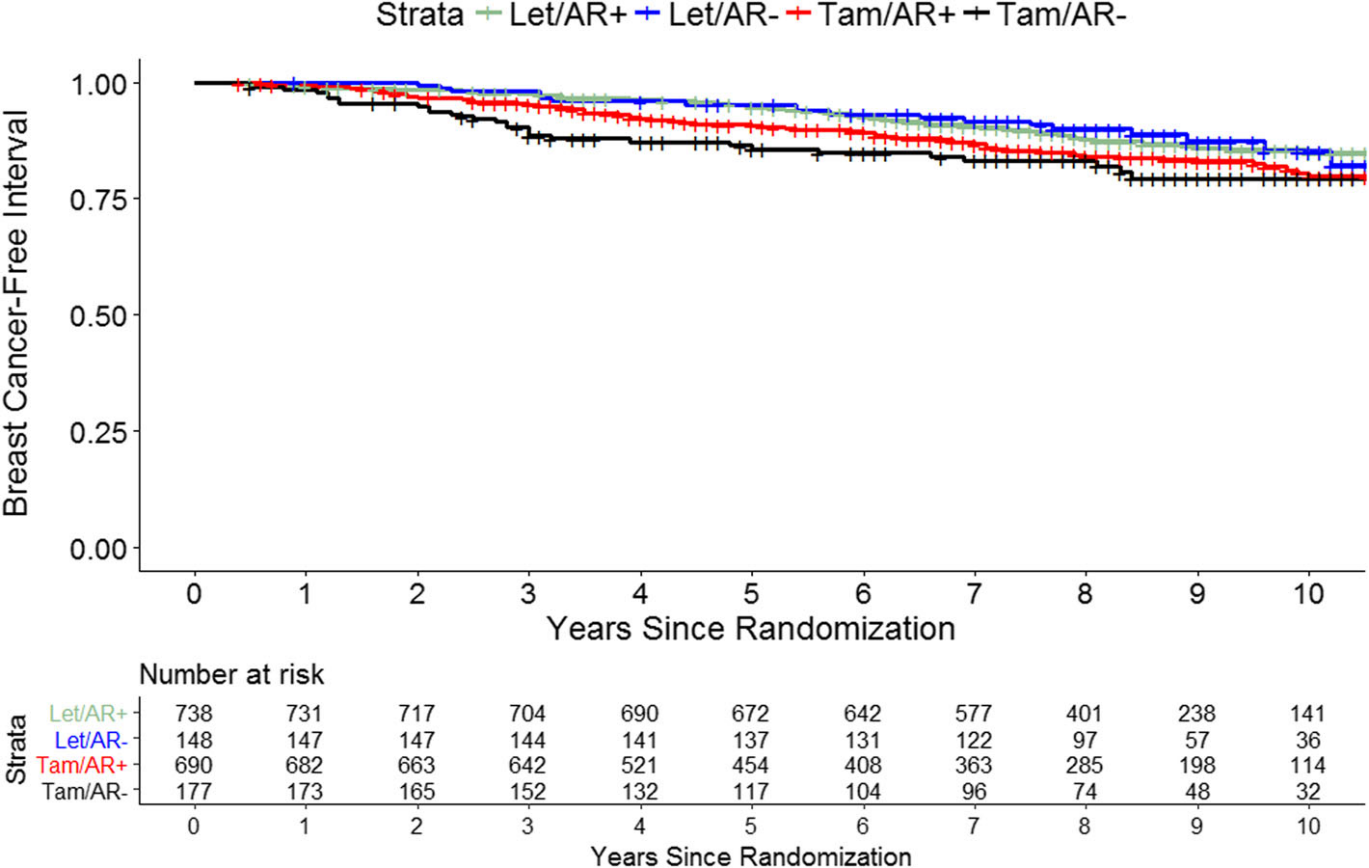
Kaplan-Meier estimate of breast cancer-free interval by cross-classified AR expression and treatment in monotherapy population. AR expression is defined as ≥ 1% positive, < 1% negative

In the Kaplan-Meier analysis in the monotherapy population (*n* = 1753), BCFI was better for those assigned to letrozole than tamoxifen, regardless of tumor AR expression (AR+ *p* = 0.02 from log-rank test; AR− *p* = 0.04) (Fig. 3). Among those receiving letrozole monotherapy, 5-year BCFI was 94.7% (95% CI 93.1–96.4%) for women with AR+ tumors and 95.2% (95% CI 91.8–98.7%) for women with AR− tumors, while among those receiving tamoxifen, 5-year BCFI was 90.7% (95% CI 88.4–93.0%) for women with AR+ tumors and 85.7% (95% CI 80.5–91.2%) for women with AR− tumors. Patterns were similar for DFS (Additional file 1: Fig. S2).

In multivariable-adjusted Cox models, participants assigned to letrozole monotherapy experienced significantly better BCFI compared to participants assigned to tamoxifen monotherapy (HR[Let.:Tam.] = 0.57, 95% CI 0.44–0.75, *p* < 0.001), as was found in the total trial population (Table 3). The treatment effect for BCFI was suggestively stronger among AR− breast cancers (HR[Let.:Tam.] = 0.39, 95% CI 0.21–0.72, *p* = 0.002) than among AR+ breast cancers (HR[Let.:Tam.] = 0.63, 95% CI 0.47–0.85, *p* = 0.003), though this difference was not statistically significant (p-heterogeneity = 0.16). Among those assigned to letrozole monotherapy, AR expression was associated with non-significantly worse BCFI (HR[AR+:AR−] = 1.52, 95% CI 0.88–2.62, *p* = 0.13), while among those assigned to tamoxifen, AR expression was not associated with BCFI (HR[AR+:AR−] = 0.94, 95% CI 0.62–1.43, *p* = 0.77). Similar trends were observed for DFS.

**Table 3:** Multivariable analysis of letrozole versus tamoxifen treatment effect by tumor AR expression

			All monotherapy	AR+	AR−	P-Het^c^
	Individuals	Events	HR (95% CI)	HR (95% CI)	HR (95% CI)	
Breast cancer-free interval						
Model 1^a^	1753	236	0.67 (0.52–0.87)	0.73 (0.55–0.98)	0.49 (0.27–0.87)	0.22
			*p* = 0.003	*p* = 0.03	*p* = 0.02	
Model 2^b^	1697	227	0.57 (0.44–0.75)	0.63 (0.47–0.85)	0.39 (0.21–0.72)	0.16
			*p* < 0.001	*p* = 0.003	*p* = 0.002	
Disease-free survival						
Model 1	1753	401	0.75 (0.62–0.92)	0.80 (0.64–0.99)	0.61 (0.39–0.94)	0.27
			*p* = 0.005	*p* = 0.04	*p* = 0.03	
Model 2	1697	388	0.69 (0.56–0.84)	0.73 (0.59–0.92)	0.52 (0.33–0.83)	0.19
			*p* < 0.001	*p* = 0.008	*p* = 0.005	

AR expression is defined as ≥ 1% positive, < 1% negative. This analysis is restricted to 1753 patients who received letrozole or tamoxifen monotherapy

^a^Model 1: Stratified by receipt of chemotherapy and adjusted for age at randomization, AR expression (all monotherapy analysis only)

^b^Model 2: Model 1 + adjusted for tumor size, lymph node involvement, local therapy, ER, PR, HER2, and Ki67 expression

^c^*P* value is from test of heterogeneity of treatment effect in AR+ and AR− cancers

**Fig. 4:**
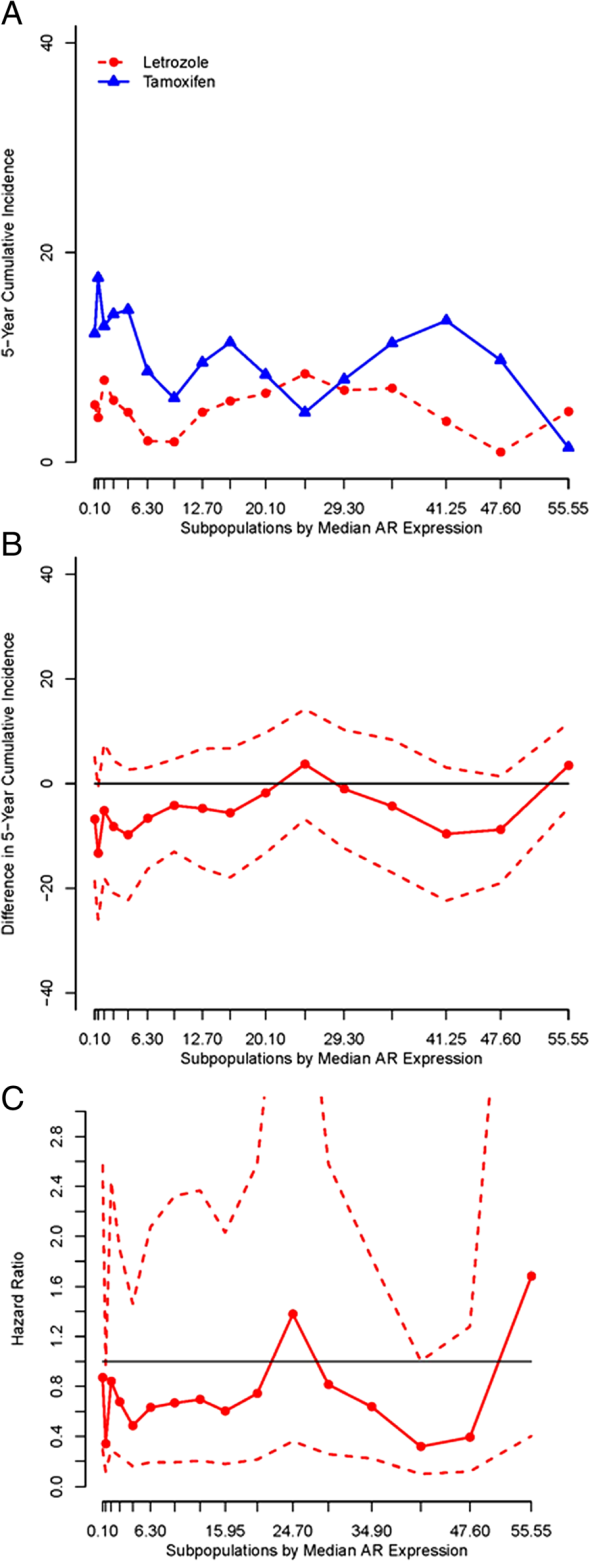
Subpopulation Treatment Effect Patten Plot (STEPP) analysis of the letrozole versus tamoxifen treatment effect. Treatment effect is defined as the 5-year BCFI in the monotherapy population (*n* = 1753). Plots show treatment effects in subpopulations denoted by median AR expression on the *x*-axis. Treatment effects are shown as **a** 5-year cumulative incidences (%), **b** 5-year cumulative incidence differences (95% CI) [Let. – Tam.], and **c** hazard ratios (95% CI) [Let.:Tam.]

STEPP analysis of the letrozole versus tamoxifen treatment effect across the continuum of AR expression indicated general superiority of letrozole monotherapy, irrespective of AR expression (Fig. 4). Five-year BCFI was generally worse for those assigned to tamoxifen monotherapy within all subpopulations of AR expression. Correspondingly, absolute and ratio measures of the treatment effect at 5 years generally favored letrozole monotherapy. There were no systematic trends in the magnitude of these effects across the continuum of AR expression.

## Discussion

We evaluated AR as a prognostic and predictive marker among 3021 postmenopausal participants of the BIG 1–98 trial with early stage ER+ breast cancer. Tumor AR expression was associated with more favorable tumor features including smaller size, lower grade, lower likelihood of lymph node involvement. After adjusting for patient, tumor, and treatment factors, AR expression was not associated with breast cancer prognosis in this population. Moreover, AR expression was not a significant predictor of therapeutic response, though, suggestively, the superiority of monotherapy with letrozole relative to tamoxifen was more pronounced among women with AR−/ER+ cancers. These findings were similar for a dichotomous measure of AR expression (cut at 1%) and when evaluating the continuum of AR expression.

The androgen receptor is an emerging prognostic marker for breast cancer, with a recent meta-analysis by Bozovic-Spasojevic et al. of 13 studies (*n* = 5648 patients) finding tumor AR expression to be associated with improved DFS in the multivariate analysis (HR = 0.46, 95% CI 0.37–0.58) [12]. This association may mask differential effects of AR signaling in ER+ and ER− breast cancers [8–10]. In vitro models of AR+/ER− breast cancers have indicated that AR signaling can drive cell proliferation in these cancers [11, 32]. However, there is inconsistent evidence for a deleterious effect of AR signaling in ER− breast cancers at a population level, with epidemiologic studies finding harmful [33–35], beneficial [36, 37], and null [5, 14, 38, 39] associations. AR has also emerged as a promising therapeutic target, particularly for AR+ triple-negative breast cancers [40]. Epidemiologic evidence generally supports an inverse association between AR expression and breast cancer mortality for ER+ cancers, though we found no association in our study. The Bozovic-Spasojevic et al. meta-analysis found improved DFS associated with AR expression in ER+ breast cancers (multivariate HR = 0.40, 95% CI 0.31–0.52, *n* = 1571 patients) [12]. Prior meta-analyses have also found inverse associations [41–43], though the addition of our study to this body of literature would attenuate overall evidence for an association. The inverse association observed in the majority of epidemiologic studies aligns with results from experimental models, where higher levels of AR were correlated with a lower rate of proliferation in MCF7 cells [44]. Treatment with enzalutamide, an AR antagonist, abrogated androgen-driven proliferation in MCF7 cells and xenographs [22]. However, blockade of androgen synthesis with abiraterone acetate plus prednisone did not improve progression-free survival relative to treatment with exemestane in a phase II trial among 297 patients with ER+ metastatic breast cancer [45]. Cistrome analyses have found that the AR and ER may compete to occupy estrogen response elements, with bound AR-reducing cellular proliferation [13, 46]. Alternatively, AR may have unique binding sites at ER target genes through which it could directly inhibit transcription or it may indirectly inhibit transcription through competition for co-regulators [10].

In contrast to our own study, several prior observational studies have found AR to be a predictive marker for endocrine therapy response in ER+ breast cancers. However, this evidence is conflicting and primarily relies on univariate analyses. In the largest study to date (*n* = 938), AR expression was associated with improved disease-specific survival for women receiving endocrine therapy and chemotherapy, but not endocrine therapy alone [47]. In contrast, in a separate cohort of 119 women with ER+ breast cancer, endocrine therapy alone was associated with better outcomes [21]. There is also evidence that AR signaling could predict endocrine therapy resistance. An AR:ER ratio ≥ 2 was associated with an increased rate of tamoxifen failure in a cohort of 192 women with ER+ breast cancer, though these tumors may be more receptive to treatment with AR-antagonists [22]. Likewise, an effect of AR expression on tamoxifen resistance has been observed in in vitro models, potentially mediated through activation of epidermal growth factor receptor signaling pathways [48, 49]. However, a study among 590 women found that AR did not predict tamoxifen response in AR+/ER+ cancers, though it did suggest a benefit to tamoxifen treatment for AR+/ER− cancers [33]. Finally, a recent study conducted among 102 patients receiving endocrine therapies for ER+ breast cancers also found no association between AR expression and treatment response [50].

We found no association between tumor AR expression and response to tamoxifen among postmenopausal women with ER+ breast cancers. We had postulated that AR expression would lead to enhanced response to letrozole via increased AR activity that would dampen ER signaling; however, our findings do not support this hypothesis. We observed that AR expression was suggestively but non-significantly associated with a higher rate of recurrence among those assigned to receive letrozole. Despite the non-significantly lower efficacy of letrozole in AR−/ER+ breast cancers, survival outcomes among those receiving letrozole monotherapy were superior to those among individuals receiving tamoxifen regardless of tumor AR expression. As such, we found no evidence that tumor AR expression should be considered in the selection of adjuvant endocrine therapy.

This study represents the largest and most comprehensive evaluation of AR expression as a predictor of endocrine therapy response to date. In particular, the randomized design allows for better assessment of therapeutic effects relative to the observational designs used in prior studies. This setting also allows for careful adjustment for tumor features that influence prognosis. However, lifestyle factors that influence breast cancer prognosis, such as body mass index, were not measured in the BIG 1–98 population. Additional strengths of this analysis include the use of central pathology assessment of AR and other tumor markers, as well as the evaluation of a continuous measure of AR expression. The optimal cut-point that maximizes clinical utility of AR has not yet been identified, though recent studies suggest that it may be greater than 1% or 10%—the most common cut-points used to date [7, 13, 51, 52]. Ricciardelli and colleagues found that neither the 1% or 10% cut-points were robustly prognostic, while a cut-point of 78% maximized sensitivity and specificity for predicting breast cancer survival [7]. Likewise, multiple antibodies have been used to measure AR protein expression, but the AR441 antibody has been used in the majority of studies evaluating AR as a prognostic marker for breast cancer [7]. AR expression was only assessable for a subset of the BIG 1–98 trial population. Patients who enrolled early into the trial were less likely to have tumor tissue submitted for tumor marker analyses. This contributes to some imbalances in tumor characteristics between the populations with and without assessable AR expression. BCFI is superior in the AR analytic population than in the total trial population, and the letrozole versus tamoxifen treatment effect is also stronger in this analytic population [17]. An additional limitation of our study is that due to large differences in the distributions of AR and ER expression in our data, we were unable to rigorously assess the prognostic and predictive value of previously identified cut-points, 0.87 and 2.0, of the AR:ER ratio [7, 22]. Finally, these findings are pertinent to the population of postmenopausal women with early stage ER+ breast cancer receiving letrozole and tamoxifen and may not necessarily reflect the influence of AR expression on the efficacy of other endocrine therapies.

## Conclusions

AR protein expression was not associated with prognosis in this population of postmenopausal women with early stage ER+ breast cancer, nor was it predictive of response to tamoxifen or letrozole. These findings do not support consideration of tumor AR expression in the choice of initial adjuvant endocrine therapy.

## Additional file

Additional file 1: Supplemental results containing: 1) a table comparing trial participants for whom AR expression was and was not assessable; 2) a Kaplan-Meier curve comparing disease-free survival by tumor AR expression; 3) a Kaplan-Meier curve comparing disease-free survival by cross-classified tumor AR expression and treatment assignment. Table S1 Hypothesis testing for differences in distributions between tumors with and without assessable AR used chi-square, Wilcoxon rank sum and log-rank tests for categorical, continuous and time-to-event endpoints, respectively. Design variables (two- or four-arm randomization period and treatment assignment) were not compared. Individuals with missing data were excluded when performing hypothesis tests for continuous variables. (DOCX 53 kb)

## Abbreviations

AR: Androgen receptor
BCFI: Breast cancer-free interval
BIG 1–98: Breast International Group Trial 1–98
CI: Confidence interval
DFS: Disease-free survival
ER: Estrogen receptor
HER2: Human epidermal growth factor 2
HR: Hazard ratio
IHC: Immunohistochemistry
IQR: Interquartile range
PR: Progesterone receptor
SERM: Selective estrogen receptor modulator
STEPP: Subpopulation treatment effect pattern plot
TMA: Tissue microarray

## References

[1] Burstein HJ, Prestrud AA, Seidenfeld J, Anderson H, Buchholz TA, Davidson NE, et al. American Society of Clinical Oncology clinical practice guideline: update on adjuvant endocrine therapy for women with hormone receptor-positive breast cancer. J Clin Oncol. 2010;28(23):3784–96. 10.1200/JCO.2009.26.3756 PMC5569672 20625130

[2] Early Breast Cancer Trialists’ Collaborative Group (2005). Effects of chemotherapy and hormonal therapy for early breast cancer on recurrence and 15-year survival: an overview of the randomised trials. Lancet.

[3] Collins LC, Cole KS, Marotti JD, Hu R, Schnitt SJ, Tamimi RM (2011). Androgen receptor expression in breast cancer in relation to molecular phenotype: results from the Nurses’ Health Study. Mod Pathol.

[4] Dimitrakakis C, Zhou J, Bondy CA (2002). Androgens and mammary growth and neoplasia. Fertil Steril.

[5] Gonzalez LO, Corte MD, Vazquez J, Junquera S, Sanchez R, Alvarez AC (2008). Androgen receptor expresion in breast cancer: relationship with clinicopathological characteristics of the tumors, prognosis, and expression of metalloproteases and their inhibitors. BMC Cancer.

[6] Niemeier LA, Dabbs DJ, Beriwal S, Striebel JM, Bhargava R (2010). Androgen receptor in breast cancer: expression in estrogen receptor-positive tumors and in estrogen receptor-negative tumors with apocrine differentiation. Mod Pathol.

[7] Ricciardelli C, Bianco-Miotto T, Jindal S, Butler LM, Leung S, McNeil CM (2018). The magnitude of androgen receptor positivity in breast cancer is critical for reliable prediction of disease outcome. Clin Cancer Res.

[8] Majumder A, Singh M, Tyagi SC (2017). Post-menopausal breast cancer: from estrogen to androgen receptor. Oncotarget.

[9] Hickey TE, Robinson JL, Carroll JS, Tilley WD (2012). Minireview: the androgen receptor in breast tissues: growth inhibitor, tumor suppressor, oncogene?. Mol Endocrinol.

[10] Lim E, Ni M, Hazra A, Tamimi R, Brown M (2012). Elucidating the role of androgen receptor in breast cancer. Clin Investig.

[11] Doane AS, Danso M, Lal P, Donaton M, Zhang L, Hudis C (2006). An estrogen receptor-negative breast cancer subset characterized by a hormonally regulated transcriptional program and response to androgen. Oncogene..

[12] Bozovic-Spasojevic I, Zardavas D, Brohee S, Ameye L, Fumagalli D, Ades F (2017). The prognostic role of androgen receptor in patients with early-stage breast cancer: a meta-analysis of clinical and gene expression data. Clin Cancer Res.

[13] Peters AA, Buchanan G, Ricciardelli C, Bianco-Miotto T, Centenera MM, Harris JM (2009). Androgen receptor inhibits estrogen receptor-alpha activity and is prognostic in breast cancer. Cancer Res.

[14] Elebro K, Bendahl PO, Jernstrom H, Borgquist S. Androgen receptor expression and breast cancer mortality in a population-based prospective cohort. Breast Cancer Res Treat. 2017;165(3):645–57. 10.1007/s10549-017-4343-0 PMC5602002 28643022

[15] Giobbie-Hurder A, Price KN, Gelber RD (2009). Design, conduct, and analyses of Breast International Group (BIG) 1–98: a randomized, double-blind, phase-III study comparing letrozole and tamoxifen as adjuvant endocrine therapy for postmenopausal women with receptor-positive, early breast cancer. Clin Trials.

[16] Thurlimann B, Keshaviah A, Coates AS, Mouridsen H, Mauriac L, Forbes JF (2005). A comparison of letrozole and tamoxifen in postmenopausal women with early breast cancer. N Engl J Med.

[17] Regan MM, Neven P, Giobbie-Hurder A, Goldhirsch A, Ejlertsen B, Mauriac L (2011). Assessment of letrozole and tamoxifen alone and in sequence for postmenopausal women with steroid hormone receptor-positive breast cancer: the BIG 1-98 randomised clinical trial at 8.1 years median follow-up. Lancet Oncol.

[18] Viale G, Giobbie-Hurder A, Regan MM, Coates AS, Mastropasqua MG, Dell'Orto P, et al. Prognostic and predictive value of centrally reviewed Ki-67 labeling index in postmenopausal women with endocrine-responsive breast cancer: results from Breast International Group Trial 1-98 comparing adjuvant tamoxifen with letrozole. J Clin Oncol. 2008;26(34):5569–75. 10.1200/JCO.2008.17.0829 PMC2651094 18981464

[19] Viale G, Regan MM, Maiorano E, Mastropasqua MG, Dell'Orto P, Rasmussen BB (2007). Prognostic and predictive value of centrally reviewed expression of estrogen and progesterone receptors in a randomized trial comparing letrozole and tamoxifen adjuvant therapy for postmenopausal early breast cancer: BIG 1-98. J Clin Oncol.

[20] Rasmussen BB, Regan MM, Lykkesfeldt AE, Dell'Orto P, Del Curto B, Henriksen KL (2008). Adjuvant letrozole versus tamoxifen according to centrally-assessed ERBB2 status for postmenopausal women with endocrine-responsive early breast cancer: supplementary results from the BIG 1-98 randomised trial. Lancet Oncol.

[21] Park S, Park HS, Koo JS, Yang WI, Kim SI, Park BW (2012). Higher expression of androgen receptor is a significant predictor for better endocrine-responsiveness in estrogen receptor-positive breast cancers. Breast Cancer Res Treat.

[22] Cochrane DR, Bernales S, Jacobsen BM, Cittelly DM, Howe EN, D'Amato NC (2014). Role of the androgen receptor in breast cancer and preclinical analysis of enzalutamide. Breast Cancer Res.

[23] Kennedy BJ (1958). Fluoxymesterone therapy in advanced breast cancer. N Engl J Med.

[24] Goldenberg IS (1964). Testosterone propionate therapy in breast cancer. Jama.

[25] Macedo LF, Guo Z, Tilghman SL, Sabnis GJ, Qiu Y, Brodie A (2006). Role of androgens on MCF-7 breast cancer cell growth and on the inhibitory effect of letrozole. Cancer Res.

[26] Hudis CA, Barlow WE, Costantino JP, Gray RJ, Pritchard KI, Chapman JA (2007). Proposal for standardized definitions for efficacy end points in adjuvant breast cancer trials: the STEEP system. J Clin Oncol.

[27] Durrleman S, Simon R (1989). Flexible regression models with cubic splines. Stat Med.

[28] Lazar AA, Cole BF, Bonetti M, Gelber RD (2010). Evaluation of treatment-effect heterogeneity using biomarkers measured on a continuous scale: subpopulation treatment effect pattern plot. J Clin Oncol.

[29] Yip WK, Bonetti M, Cole BF, Barcella W, Wang XV, Lazar A (2016). Subpopulation Treatment Effect Pattern Plot (STEPP) analysis for continuous, binary, and count outcomes. Clin Trials.

[30] Schoenfeld DA (1983). Sample-size formula for the proportional-hazards regression model. Biometrics..

[31] Schmoor C, Sauerbrei W, Schumacher M (2000). Sample size considerations for the evaluation of prognostic factors in survival analysis. Stat Med.

[32] Ni M, Chen Y, Lim E, Wimberly H, Bailey ST, Imai Y (2011). Targeting androgen receptor in estrogen receptor-negative breast cancer. Cancer Cell.

[33] Hilborn E, Gacic J, Fornander T, Nordenskjold B, Stal O, Jansson A (2016). Androgen receptor expression predicts beneficial tamoxifen response in oestrogen receptor-alpha-negative breast cancer. Br J Cancer.

[34] Elebro K, Borgquist S, Simonsson M, Markkula A, Jirstrom K, Ingvar C (2015). Combined androgen and estrogen receptor status in breast cancer: treatment prediction and prognosis in a population-based prospective cohort. Clin Cancer Res.

[35] Kensler KH, Poole EM, Heng YJ, Collins LC, Glass B, Beck AH, et al. Androgen receptor expression and breast cancer survival: results from the nurses’ health studies. J Natl Cancer Inst. 2018. Article is in press. 10.1093/jnci/djy173 PMC6624168 30445651

[36] Agoff SN, Swanson PE, Linden H, Hawes SE, Lawton TJ (2003). Androgen receptor expression in estrogen receptor-negative breast cancer. Immunohistochemical, clinical, and prognostic associations. Am J Clin Pathol.

[37] Luo X, Shi YX, Li ZM, Jiang WQ (2010). Expression and clinical significance of androgen receptor in triple negative breast cancer. Chin J Cancer.

[38] Loibl S, Muller BM, von Minckwitz G, Schwabe M, Roller M, Darb-Esfahani S (2011). Androgen receptor expression in primary breast cancer and its predictive and prognostic value in patients treated with neoadjuvant chemotherapy. Breast Cancer Res Treat.

[39] Gonzalez-Angulo AM, Stemke-Hale K, Palla SL, Carey M, Agarwal R, Meric-Berstam F (2009). Androgen receptor levels and association with PIK3CA mutations and prognosis in breast cancer. Clin Cancer Res.

[40] Kono M, Fujii T, Lim B, Karuturi MS, Tripathy D, Ueno NT (2017). Androgen receptor function and androgen receptor-targeted therapies in breast cancer: a review. JAMA Oncol.

[41] Vera-Badillo FE, Templeton AJ, de Gouveia P, Diaz-Padilla I, Bedard PL, Al-Mubarak M (2014). Androgen receptor expression and outcomes in early breast cancer: a systematic review and meta-analysis. J Natl Cancer Inst.

[42] Qu Q, Mao Y, Fei XC, Shen KW (2013). The impact of androgen receptor expression on breast cancer survival: a retrospective study and meta-analysis. PLoS One.

[43] Kim Y, Jae E, Yoon M (2015). Influence of androgen receptor expression on the survival outcomes in breast cancer: a meta-analysis. J Breast Cancer.

[44] Szelei J, Jimenez J, Soto AM, Luizzi MF, Sonnenschein C (1997). Androgen-induced inhibition of proliferation in human breast cancer MCF7 cells transfected with androgen receptor. Endocrinology.

[45] O'Shaughnessy J, Campone M, Brain E, Neven P, Hayes D, Bondarenko I (2016). Abiraterone acetate, exemestane or the combination in postmenopausal patients with estrogen receptor-positive metastatic breast cancer. Ann Oncology.

[46] Need EF, Selth LA, Harris TJ, Birrell SN, Tilley WD, Buchanan G (2012). Research resource: interplay between the genomic and transcriptional networks of androgen receptor and estrogen receptor alpha in luminal breast cancer cells. Mol Endocrinol.

[47] Castellano I, Allia E, Accortanzo V, Vandone AM, Chiusa L, Arisio R (2010). Androgen receptor expression is a significant prognostic factor in estrogen receptor positive breast cancers. Breast Cancer Res Treat.

[48] De Amicis F, Thirugnansampanthan J, Cui Y, Selever J, Beyer A, Parra I (2010). Androgen receptor overexpression induces tamoxifen resistance in human breast cancer cells. Breast Cancer Res Treat.

[49] Ciupek A, Rechoum Y, Gu G, Gelsomino L, Beyer AR, Brusco L (2015). Androgen receptor promotes tamoxifen agonist activity by activation of EGFR in ERalpha-positive breast cancer. Breast Cancer Res Treat.

[50] Bronte G, Rocca A, Ravaioli S, Puccetti M, Tumedei MM, Scarpi E (2018). Androgen receptor in advanced breast cancer: is it useful to predict the efficacy of anti-estrogen therapy?. BMC Cancer.

[51] Tokunaga E, Hisamatsu Y, Taketani K, Yamashita N, Akiyoshi S, Okada S (2013). Differential impact of the expression of the androgen receptor by age in estrogen receptor-positive breast cancer. Cancer medicine.

[52] Jiang HS, Kuang XY, Sun WL, Xu Y, Zheng YZ, Liu YR (2016). Androgen receptor expression predicts different clinical outcomes for breast cancer patients stratified by hormone receptor status. Oncotarget..

