# Supplementary material for: Prognostic and predictive value of androgen receptor expression in postmenopausal women with estrogen receptor-positive breast cancer: results from the Breast International Group Trial 1–98
Source: Breast Cancer Res. 2019 Feb 22;21:30. doi: 10.1186/s13058-019-1118-z (PMC6387478; doi:10.1186/s13058-019-1118-z)
Supplement: Supplementary file 1 — Supplemental results containing: 1) a table comparing trial participants for whom AR expression was and was not assessable; 2) a Kaplan-Meier curve comparing disease-free survival by tumor AR expression; 3) a Kaplan-Meier curve comparing disease-free survival by cross-classified tumor AR expression and treatment assignment. Table S1 Hypothesis testing for differences in distributions between tumors with and without assessable AR used chi-square, Wilcoxon rank sum and log-rank tests for categorical, continuous and time-to-event endpoints, respectively. Design variables (two- or four-arm randomization period and treatment assignment) were not compared. Individuals with missing data were excluded when performing hypothesis tests for continuous variables. (DOCX 53 kb) [file 13058_2019_1118_MOESM1_ESM.docx]

**Additional file 1: Table S1**. Comparison of individuals in AR analytic population to the rest of the BIG 1-98 study population

| Characteristic | Assessable AR  (n=3,103) | No Assessable AR  (n=4,907) | P-Value* |
| --- | --- | --- | --- |
| Treatment Assignment, % |  |  | -- |
| Tamoxifen | 21 | 19 |  |
| Letrozole | 21 | 18 |  |
| Tamoxifen 🡪 Letrozole | 21 | 18 |  |
| Letrozole 🡪 Tamoxifen | 21 | 18 |  |
| Tamoxifen (2 arm period) | 8 | 14 |  |
| Letrozole (2 arm period) | 8 | 13 |  |
| Received Chemotherapy, % | 30 | 22 | <0.001 |
| Local Therapy, % |  |  | <0.001 |
| Breast Conserving Surgery with Radiation Therapy | 62 | 48 |  |
| Breast Conserving Surgery without Radiation Therapy | 4 | 2 |  |
| Mastectomy with Radiation Therapy | 13 | 21 |  |
| Mastectomy without Radiation Therapy | 20 | 28 |  |
| Age at Randomization, mean(SD) | 62 (8) | 62 (8) | 0.08 |
| Tumor Size, % |  |  | 0.005 |
| ≤ 2cm | 64 | 61 |  |
| >2 - <5cm | 32 | 34 |  |
| ≥ 5cm | 4 | 4 |  |
| Missing | 1 | 1 |  |
| Number of Lymph Nodes Positive, % |  |  | <0.001 |
| 0 | 62 | 55 |  |
| 1-3 | 27 | 30 |  |
| 4-9 | 7 | 10 |  |
| 10+ | 3 | 5 |  |
| Not evaluable | 1 | 1 |  |
| Tumor Grade, % |  |  | <0.001 |
| 1 | 20 | 21 |  |
| 2 | 55 | 55 |  |
| 3 | 24 | 20 |  |
| Missing | 0 | 4 |  |
| ER Expression (%), median (25^th^-75^th^ percentile) | 95 (90-99) | 90 (80-99) | 0.11 |
| Missing, n | 52 | 1679 |  |
| PR Expression (%), median (25^th^-75^th^ percentile) | 70 (10-90) | 70 (10-90) | 0.001 |
| Missing, n | 66 | 1683 |  |
| Ki67 Expression (%), median (25^th^-75^th^ percentile) | 12 (6-19) | 12 (7-18) | 0.33 |
| Missing, n | 130 | 1903 |  |
| HER2 Status, % |  |  | <0.001 |
| Positive | 6 | 5 |  |
| Negative | 93 | 62 |  |
| Missing | 1 | 33 |  |
| Follow-up (years), median (25^th^-75^th^ percentile) | 8.1 (7.3-9.1) | 8.2 (7.3-9.9) | -- |
| Breast Cancer-free Interval |  |  | <0.001 |
| Events, n | 433 | 905 |  |
| 8-year BCFI % | 86.6 | 82.4 |  |
| Disease-free Survival |  |  | <0.001 |
| Events, n (%) | 701 | 1373 |  |
| 8-year DFS % | 78.9 | 74.1 |  |

*****Hypothesis testing for differences in distributions between tumors with and without assessable AR used chi-square, Wilcoxon rank sum and log-rank tests for categorical, continuous and time-to-event endpoints, respectively. Design variables (2- or 4-arm randomization period and treatment assignment) were not compared. Individuals with missing data were excluded when performing hypothesis tests for continuous variables.

**Figure S1**. Kaplan-Meier survival curve for disease-free survival by tumor AR expression. AR expression is defined as ≥1% positive, <1% negative. Disease-free survival at 5 years was 88.2% (95% CI 87.0%-89.5%) for women with AR+ tumors and 86.8% (95% CI 84.0%-89.7%) for women with AR- tumors. The p-value from log rank test is 0.35.

**
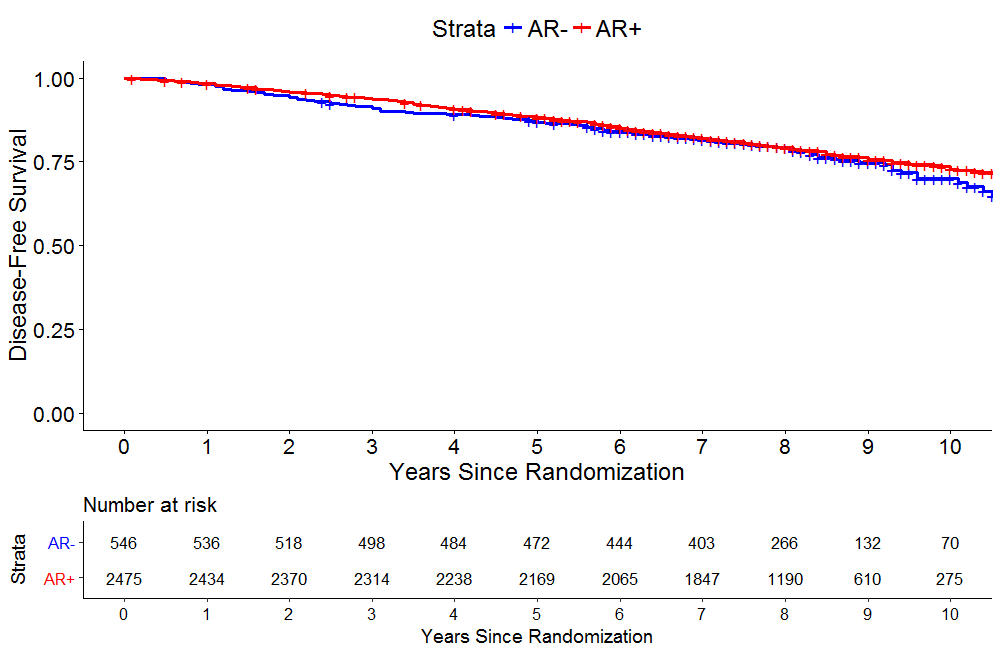
**

**Figure S2**. Kaplan-Meier survival curve for disease-free survival by cross-classified AR expression and treatment in the monotherapy population. AR expression is defined as ≥1% positive, <1% negative. Among those receiving letrozole, disease-free survival at 5 years was 89.4% (95% CI 87.2%-91.6%) for women with AR+ tumors and 92.6% (95% CI 88.4%-96.9%) for women with AR- tumors. Among those receiving tamoxifen, disease-free survival at 5 years was 85.4% (95% CI 82.7%-88.2%) for women with AR+ tumors and 80.9% (95% CI 75.2%-87.0%) for women with AR- tumors.

**
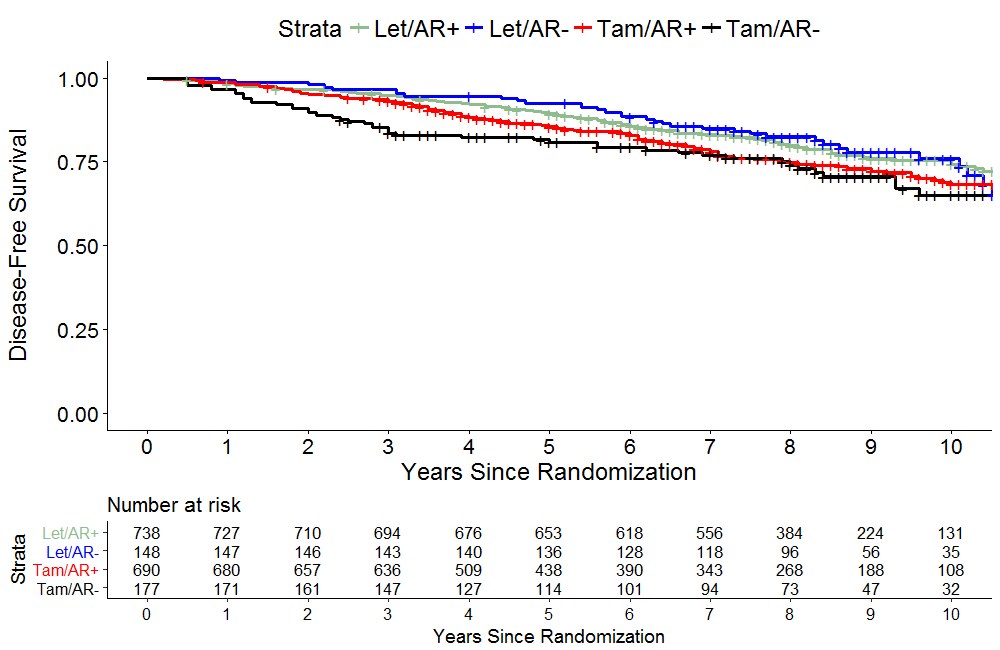
**
